# Older Veteran Digital Disparities: Examining the Potential for Solutions Within Social Networks

**DOI:** 10.2196/jmir.6385

**Published:** 2016-11-23

**Authors:** Tana M Luger, Timothy P Hogan, Lorilei M Richardson, Lisa Cioffari-Bailiff, Kimberly Harvey, Thomas K Houston

**Affiliations:** ^1^ Pitzer College Claremont, CA United States; ^2^ Edith Nourse Rogers Memorial Veterans Hospital Bedford, MA United States; ^3^ Department of Quantitative Health Sciences University of Massachusetts Medical School Worcester, MA United States; ^4^ Department of Graduate Nursing University of Massachusetts Medical School Worcester, MA United States; ^5^ VA Boston Healthcare System Boston, MA United States

**Keywords:** Internet, digital divide, social network, Veterans

## Abstract

**Background:**

Older adults typically have less access to the Internet than other age groups, and older Veterans may use the Internet even less due to economic and geographic reasons.

**Objective:**

To explore solutions to this problem, our study examined older Veterans’ reported ability to access technology through their close social ties.

**Methods:**

Data were collected via mail survey from a sample of Veterans aged 65 years and older (N=266).

**Results:**

Nearly half (44.0%, 117/266) of the sample reported having no Internet access. Yet, among those without current access, older Veterans reported having a median of 5 (IQR 7) close social ties with home Internet access. These older Veterans also reported that they would feel comfortable asking a median of 2 (IQR 4) social ties for help to access the Internet, and that a median of 2 (IQR 4) social ties would directly access the Internet for the older Veteran to help with health management.

**Conclusions:**

Findings suggest that even older Veterans without current Internet access have at least two social ties with home Internet who could be called upon for technology support. Thus, older Veterans may be willing to call upon these “surrogate seekers” for technology assistance and support in health management. This has implications for the digital divide, technology design, and health care policy.

## Introduction

It is well documented that a “digital divide” exists whereby older adults are less likely to access the Internet than other age groups [1-5]. Recent statistics suggest that 59% of US adults aged 65 years or older use the Internet, compared to 86% of all US adults older than 18 years [6]. There are a number of reasons cited for this discrepancy in the literature [4-7]. For example, older adults may have insufficient digital skills to use the Internet, inadequate finances to purchase equipment or an Internet service, or perceive limited personal benefit to using the Internet and other technologies [4,7]. Yet, there is variation of use even among the elderly; older adults who do use the Internet are typically wealthier, more educated, and reside in more urban areas compared to older adult nonusers [6,8]. In fact, there appears to be two burgeoning groups of older adults in the United States: younger, wealthier technology adopters and their older, less affluent counterparts [6]. Veterans older than age 65 years, who represent approximately 46% of the US Veteran population [9], typically earn a lower income than their civilian counterparts [10-12], which may point to an even greater disparity in Internet use. Thus, this paper aims to examine the digital divide in an aging group of Veterans to begin to understand technology adoption in this vulnerable group.

The digital divide is particularly concerning due to the fact that health information technologies (HIT), such as Web-based patient portals, mobile apps, or telehealth systems, are increasingly implemented to provide patients with improved access to their health care providers and self-management resources [13-16]. Such technologies can offer patients and health care providers real-time information about health conditions [17-19], enhance patient-provider communication by allowing information sharing and electronic messaging between involved parties [19,20], and improve patient outcomes by allowing patients access to health information and tools which can aid problem solving, decision making, and goal setting [18,19]. The Veteran’s Health Administration (VHA) has been a leader in developing HIT to supplement and enhance face-to-face health care visits, including the MyHealth*e*Vet patient portal which features health information and asynchronous secure messaging, the Care Coordination Telehealth Program to provide in-home remote monitoring and consults for chronic disease, and VHA mobile apps that range from weight management coaching to a summary of personal VHA medical information [15,21,22]. With many health care systems turning to technology to facilitate information sharing, communication, and remote monitoring critical to continuity of care, patients without access to technology may find themselves excluded from these promising innovations.

As previously mentioned, older US Veterans are a particularly useful group in which to study barriers to technology access because the social and economic factors that contribute to digital disparities are more common among Veterans. For example, Veterans typically earn a lower income and live in more rural areas than their civilian counterparts [10-12], both of which are associated with a lower rate of technology adoption. In addition, Veterans cope with more health conditions and report poorer health than civilians [10-12], indicating an even greater need for the support of HIT. We examine a group of older Veterans who have yet to adopt the VHA’s patient portal in order to begin to explore the practical barriers to using health information technology in this population.

Many older Veterans have family or informal caregivers who help them to manage their health care [23-26]. Thus, an examination of older Veterans is incomplete without consideration of their social context. Research on social networks suggests that people are connected to one another by strong ties (eg, family, close friends) and weak ties (eg, coworkers, acquaintances) [27-30]. For older adults, the presence of social ties has been associated with a lower risk of mortality [31], fewer depressive symptoms [32], and a reduced rate of cognitive decline [33-35]. Conversely, a lack of ties has been associated with poorer self-rated health [36], higher blood pressure [37], and higher systemic inflammation [37]. Social ties play a role in health and behavior by (1) providing emotional, tangible, or informational support; (2) reinforcing group attitudes and social norms for behaviors; (3) promoting social engagement and participation; and (4) providing access to material resources [27,30,38].

As health care systems such as VHA promote use of patient-facing technology, patients without access to a resource such as the Internet may find themselves at a disadvantage. New solutions for linking patients to technology are needed to ensure that the digital divide does not inadvertently widen, especially in the health arena. Our study examined a sample of older Veterans in order to study the digital divide among a population of lower socioeconomic status older adults with complex health needs, and quantify whether older Veterans might gain access to technology through their social contacts for the concrete purpose of managing their health. Thus, we conducted a 1-year, VHA-funded pilot study to

1. Describe access to and use of technology among a purposeful sample of older Veterans, and

2. Examine older Veterans’ reported ability to access the Internet through their social ties for the purpose of health management.

## Methods

### Setting and Sample

The sample was drawn from the VHA system of electronic health records available through the VHA Corporate Data Warehouse. Veterans of the US armed services aged 65 years and older who had at least two outpatient care visits at a VHA facility between October 1, 2012 and August 1, 2013 were eligible for inclusion. Given the potential for cognitive deficits to influence technology use and shape informal caregiving needs in ways that would not be analogous to other participants, older Veterans with a documented diagnosis of dementia were excluded from participation. Older Veterans were also excluded from the cohort if they were already registered with VHA’s personal health record, MyHealth*e*Vet. This criterion was used to ensure a sufficient number of older Veterans with limited computer skills or interest among the sample.

Eligible older Veterans were purposefully sampled according to race (white, black, or Hispanic/Latino), marital status (married or single/divorced/widowed), and US geographic location (Northeast, Midwest, South, or West) to allow for a sample of varied demographics. A total of 1500 eligible older Veterans were randomly identified as potential participants and their contact information (name and address) was obtained from the electronic health record. The study was approved by the Institutional Review Board at the Edith Nourse Rogers Veterans Hospital in Bedford, MA.

### Survey Procedure and Rates of Response

Survey items were drawn from three previously fielded US telephone or mail surveys examining device ownership, technology use, and health in the civilian population. The surveys included the Computer-Email-Web Fluency Scale [39], Internet Use Among Midlife and Older Adults [40], and the Pew Research Center’s Internet Project Tracking Survey [41]. Items related to Internet access through social ties were developed by the research team. The survey was piloted by a small convenience sample (N=7) who provided written responses regarding the format, organization, and readability of the items. Items were then refined by the research team to produce the final mail survey.

To encourage response to the survey, potential Veteran participants were mailed an introductory letter that explained the purpose and procedures of the study. Two weeks after the initial letter, participants received a token incentive (a miniature calendar), a paper copy of the survey, and a stamped return envelope through the mail. Surveys were fielded between December 2013 and July 2014. A total of 121 surveys were returned as a result of outdated contact information or patient death. In total, 19.29% (266/1379) of the sample completed the survey and were included in analyses.

### Survey Variables

#### Demographics

Participants responded to several questions about their race, education, annual household income, marital status, and self-reported health status. Information on age, gender, race, geographic location (rural/urban), and number of chronic conditions was obtained from the VHA Corporate Data Warehouse system of records to compare with self-reported demographics and to supplement survey data. Finally, participants responded to two items related to health literacy that assessed the extent to which they needed help reading hospital materials and their confidence in filling out medical forms for themselves [42].

#### Technology Engagement

Participants reported information about their technology engagement by responding to items regarding (1) technological devices used in the past month, (2) methods of Internet access (eg, home computer, library, senior center), (3) Internet experience (ie, comfort with the Internet, typical activities, average use per day), and (4) cellular phone use for text messaging. For each of these questions, participants were instructed to mark all response options that applied to their personal ownership, use, or interest. As a result, the frequencies that we report are not mutually exclusive and represent the percentage of participants who endorsed each response option.

#### Self-Reports of Social Ties

Participants were asked to report about three distinct categories of social ties: adult children, extended family, and friends. Participants estimated (1) the total number of ties with whom they had spoken in the past 4 weeks, (2) the number of ties with home Internet access (eg, through a computer or smartphone), (3) the number of ties who would allow the older Veteran to use their device to access the Internet for the purpose of health management, (4) the number of ties whom the Veteran would feel comfortable asking for help to use the Internet for health management, and (5) the number of ties who would be willing to use the Internet on behalf of the older Veteran for health management.

### Analyses

We used SPSS version 20 to calculate frequencies and measures of central tendency to describe the older Veteran sample and their personal access to and use of technology and the Internet. Participants marked all response options that applied to their personal ownership and use of technology. As a result, the frequencies that we report are not mutually exclusive and represent the percentage of participants who endorsed each response option. Then, participants were characterized based on their response to the following question: “How do you currently access the Internet?” Veterans who reported having no Internet access were compared to Veterans with current Internet access (eg, via home computer, library, or senior center) by utilizing chi-square tests for independence adjusted by Bonferroni correction and post hoc *z* tests. Listwise deletion was employed to handle missing responses. Next, we used descriptive statistics to examine older Veterans’ perceived ability to access the Internet through their social ties. We summed participant responses for each social tie category (adult children, extended family, and friends) to create an overall score for each social tie survey item (see Survey Variables in Methods section). We report social tie data for the entire sample as well as a focus on those Veterans without Internet access. To further investigate participants without Internet access, we grouped these older Veterans according to their reported number of ties with Internet access (no ties, at least one tie, two or more ties).

## Results

### Veteran Respondent Characteristics

Veteran respondents were predominantly male (95.9%, 255/266) and white (77.4%, 206/266; black: 14.3%, 38/266; Hispanic/Latino: 8.3%, 22/266) with a mean age of 75.7 (SD 7.9, range 65-96) years. A quarter of respondents (25.6%, 68/266) resided in rural areas, and 59.0% (157/266) were married or partnered. One-third had a high school education or less (31.6%, 84/266), and 80.5% (214/266) earned an income of less than US $45,000 annually. Nearly half (45.5%, 121/266) of respondents reported being in good health. Respondents were diagnosed with mean of 3.4 (SD 4.3, range 0-17) chronic conditions.

### Technology Access and Use

Nearly half (44.0%, 117/266) of respondents reported that they did not have access to the Internet. Veterans without Internet access were more likely to be older, unmarried, have completed less education, and earn a lower annual income than those Veterans reporting current Internet access (see Table 1). In addition, Veterans without Internet access were less likely to report being in good health and less likely to be confident in filling out medical forms without assistance, a marker of poorer health literacy.

**Table 1:** Veteran demographic characteristics compared by Internet access (N=266).

Variables		Older Veterans with current Internet access, n (%) (n=149)	Older Veterans with no Internet access, n (%) (n=117)	*P* value (*z* test)
**Age (years)**				
	65-75	89 (59.7)	41 (35.0)	<.001
	76-85	49 (32.9)	52 (44.4)	.05
	≥86	11 (7.4)	24 (20.5)	.002
**Gender**				
	Male	144 (96.6)	111 (94.9)	.47
	Female	5 (3.4)	6 (5.1)	.47
**Race/Ethnicity**				
	Black	20 (13.4)	18 (15.4)	.65
	White	117 (78.5)	89 (76.1)	.47
	Hispanic	12 (8.1)	10 (8.5)	.63
**Marital status**				
	Married/partnered	97 (65.1)	60 (51.3)	.02
	Unmarried (single, divorced, or widowed)	52 (34.9)	57 (48.7)	.02
**Rural status**				
	Urban	114 (77.6)	82 (70.1)	.24
	Rural	33 (22.4)	35 (29.9)	.15
**Education**				
	Elementary	1 (0.7)	2 (1.7)	.42
	Middle	7 (4.8)	10 (8.6)	.20
	High school	27 (18.5)	37 (31.9)	.01
	Some college/vocational	48 (32.9)	41 (35.3)	.62
	Associates	13 (8.9)	8 (6.9)	.57
	College degree	27 (18.5)	11 (9.5)	.04
	Graduate degree	23 (15.8)	7 (6.0)	.02
**Income (US $)**				
	5000-10,000	4 (3.0)	13 (12.5)	.005
	10,001-15,000	11 (7.4)	24 (23.1)	.002
	15,001-25,000	20 (13.4)	27 (26.0)	.04
	25,001-35,000	38 (25.5)	21 (20.2)	.14
	35,001-45,000	16 (10.7)	10 (9.6)	.55
	>$45,000	43 (28.9)	9 (8.7)	<.001
**Health status**				
	Very poor	2 (1.4)	6 (5.2)	.07
	Poor	8 (5.4)	13 (11.2)	.09
	Fair	48 (32.7)	49 (42.2)	.10
	Good	77 (52.4)	44 (37.9)	.02
	Excellent	12 (8.2)	4 (3.4)	.11
			
			
			
			
**Need help reading hospital materials? (health literacy)**				
	Always	14 (9.4)	22 (19.3)	.03
	Often	7 (4.7)	6 (5.3)	.87
	Sometimes	17 (11.4)	11 (9.6)	.60
	Occasionally	28 (18.8)	19 (16.7)	.60
	Never	83 (55.7)	56 (49.1)	.20
**Confident filling out medical forms? (health literacy)**				
	Not at all	13 (8.7)	23 (20.2)	.01
	A little bit	8 (5.4)	14 (12.3)	.05
	Somewhat	26 (17.4)	29 (25.4)	.14
	Quite a bit	45 (30.2)	23 (20.2)	.05
	Extremely	57 (38.3)	25 (21.9)	.003

Across the entire sample, 45.5% (121/266) of respondents reported gaining Internet access through a home computer, whereas 11.7% (31/266) of respondents gained Internet access through a smartphone or tablet. Few respondents reported gaining Internet access through community settings, such as a library (4.5%, 12/266) or senior center (1.5%, 4/266). Others reported gaining direct Internet access by using a family member’s (13.2%, 35/266) or a friend’s (3.0%, 8/266) computer.

When asked about their technology use in the past month, 66.8% (175/262) of respondents had used a cellular phone, whereas 17.6% (46/262) of respondents had used a smartphone. Among these older Veterans, 21.8% (57/262) reported sending text messages from their phone, sending texts to their children, friends, and spouse most frequently. In terms of computing devices, 37.8% (99/262) of all respondents had used a desktop computer in the past month, 20.2% (53/262) had used a laptop computer, and 9.9% (26/262) had used a tablet.

Table 2 compares the technology use of older Veterans based on their current Internet access. Veterans reporting no Internet access were also more likely to report being very uncomfortable using the Internet (χ^2^_4_=82.3, *P*<.001), and less likely to report having used a smartphone (χ^2^_1_=39.4, *P*<.001), tablet (χ^2^_4_=18.8, *P*<.001), desktop computer (χ^2^_1_=113.3, *P*<.001), or laptop computer (χ^2^_1_=43.4, *P*<.001) in the past 4 weeks than those with current access. Of note, there was no significant difference in the proportion of older Veterans using a cellular phone in the past 4 weeks based on current Internet access (χ^2^_1_=1.6, *P*=.20). However, there was a significant difference in the proportion of older Veterans who sent text messages on their cell phone (χ^2^_1_=17.2, *P*<.001); only 6.8% (8/115) without Internet access sent text messages.

**Table 2:** Technology use of Veterans compared by Internet access.

Variables		Older Veterans with current Internet access, n (%) (n=147)	Older Veterans with no Internet access, n (%) (n=95/115)^a^	*P* value (*z* test)
**Comfort using the Internet**				
	Very comfortable	54 (36.7)	5 (5.3)	.001
	Somewhat comfortable	37 (25.2)	7 (7.4)	.001
	Neither comfortable nor uncomfortable	21 (14.3)	17 (17.9)	.45
	Somewhat uncomfortable	18 (12.2)	7 (7.4)	.22
	Very uncomfortable	17 (11.6)	59 (62.1)	.001
**Devices used in the past month**				
	None	7 (4.8)	42 (36.5)	.001
	Cellular phone	103 (70.1)	72 (62.6)	.20
	Smartphone	45 (30.6)	1 (0.9)	.001
	Desktop computer	97 (66.0)	2 (1.7)	.001
	Laptop	51 (34.7)	2 (1.7)	.001
	Tablet	25 (17.0)	1 (0.9)	.001
**Sends text messages**				
	Yes	48 (32.7)	8 (7.0)	.001
	No	89 (60.5)	74 (64.3)	.53

^a^ Devices used in past month and sends text messages: n=115.

### Access Through Social Ties

**Figure 1:**
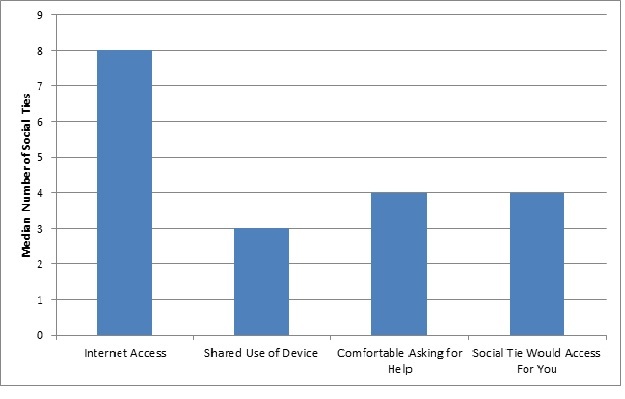
Older Veterans’ reported Internet access through social ties (n=170).

We examined respondents’ reported ability for Internet access through social ties (see Figure 1). Older Veterans reported a median of 8 (IQR 9) social ties with home Internet access when asked to consider the people that they had spoken to in the past 4 weeks. Among those social ties with Internet access, older Veterans reported that a median of 3 (IQR 8) social ties would share use of a technological device to allow them to use the Internet for the purpose of health management. Older Veterans felt comfortable asking a median of 4 (IQR 6) social ties for help to use the Internet for health management. Finally, older Veterans reported that a median of 4 (IQR 7) social ties would be willing to use the Internet for them to manage their health.

Focusing on those Veterans without Internet access (which directly represents the digital divide; n=117), we found that these older Veterans still reported a median of 5 (IQR 7) social ties with home Internet access. Similarly, those older Veterans without Internet access reported a median of 1 (IQR 4) social tie who would share use of a technological device for health management. Older Veterans without Internet access also reported feeling comfortable asking a median of 2 (IQR 4) social ties for help to use the Internet and median of 2 (IQR 4) social ties that would use the Internet for the older Veteran for the purpose of health management.

A closer examination of those older Veterans without Internet access showed that the majority (81.2%, 95/117) reported having two or more social ties with home Internet access (see Table 3). In addition, slightly more than half (54.7%, 64/117) reported having two or more ties that they would feel comfortable asking for help to use the Internet and two or more ties that would use the Internet for them (56.4%, 66/117).

**Table 3:** Proportions of reported social ties by older Veterans without Internet access (N=117).

Variables	No reported ties/missing, n (%)	1 tie, n (%)	≥2 Ties, n (%)
Social ties with home Internet access	13 (11.1)	9 (7.7)	95 (81.2)
Social ties would share use of a device	60 (51.3)	15 (12.8)	42 (35.9)
Social ties that Veteran would ask for help with Internet	35 (29.9)	18 (15.4)	64 (54.7)
Social ties would access the Internet for the Veteran	35 (29.9)	16 (13.7)	66 (56.4)

**Figure 2:**
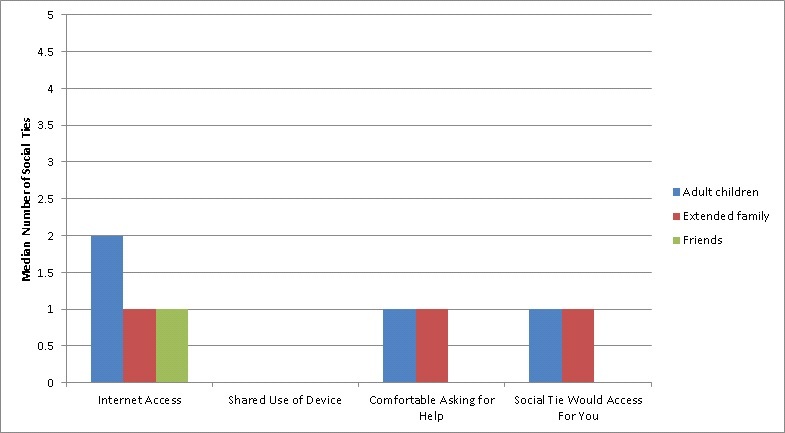
Perceived Internet access through specific types of social ties (adult children, extended family, or friends) among older Veterans without current Internet access (n=95).

Figure 2 shows the reported Internet access through social ties by specific tie category (eg, adult child, extended family member, and friend). Those older Veterans without Internet access still reported a median of 2 (IQR 4) adult children, a median of 1 (IQR 3) extended family member, and a median of 1 (IQR 2) friend with home Internet access. Furthermore, Veterans without Internet access reported a median of 1 (IQR 2) adult child and a median of 1 (IQR 2) extended family member whom the older Veteran would feel comfortable asking for help to use the Internet. Veterans without Internet access also reported a median of 1 (IQR 2) adult child and a median of 1 (IQR 2) extended family member who would use the Internet for the respondent.

## Discussion

### Principal Results

Our study investigated a sample of older Veterans to understand their personal access to and use of technology as well as their reported ability to access technology through their social ties. We chose to examine a group of older Veterans because they represent an increasingly growing proportion of the US population [9,43,44], and present with complex health care needs [10-12] that could benefit from HIT support. In addition, the social and economic barriers to technology adoption are more common in Veterans. Thus, research conducted with Veterans can suggest solutions relevant to our more vulnerable patient subgroups. Our study provides a unique opportunity to examine the technology use and social relationships of a national cohort of vulnerable older adults. Among our sample, most of who were lower income, less educated, and managing multiple chronic conditions, we found that nearly half reported having no Internet access. Yet, these older Veterans without Internet access still reported a median of 5 (IQR 7) people in their lives with home Internet access, with more than four-fifths reporting two or more social ties with access.

### The Reach of Technology Among Older Veterans

To place our sample in context, our respondents were similar in racial background, marital status, educational attainment, and income as compared to other studies of elderly Veterans [11,43] as well as the overall Veteran population [9]. Our entire sample of older Veteran respondents also had attained less education and earned less income than their civilian counterparts [45]. Thus, our study illustrates the technology use of a group typically at risk for digital disparities.

Recent surveys of civilian older adults from organizations such as the AARP and the Pew Research Center’s Internet & American Life Project, have estimated that approximately half of older adults have access to and use the Internet, and most gain access through their home computer [6,40]. Our findings corroborate these estimates with 45% of our older Veteran respondents reporting Internet access through their home computer. Compared to Pew’s estimation that 18% of older adults have adopted smartphones [6], we found similar rates of older Veterans reporting Internet access through a smartphone (18%), and that cellular phone use had been largely adopted by both older Veterans (~66%) and older civilians (77%, according to [6]). Older Veterans appear to adopt technological devices and use the Internet at similar rates as the older civilian population.

In our sample, older Veterans reporting current Internet access were younger, more educated, and wealthier than those Veterans reporting no access. These demographic predictors of Internet access (age, education, and income) are consistent with other studies of the digital divide in civilians [2,6,8,41], as well as other investigations of Veterans [46,47]. Because approximately two-thirds of our sample earned less than US $35,000 per year, it is possible that the patterns of use reported by older Veterans were driven by income rather than age. Nevertheless, recent Pew Research Center data report that among US adults who have yet to adopt the Internet, 41% are older than 65 years old, whereas 23% earn less than US $30,000 per year [48], which may indicate that age is still a strong predictor of technology adoption. Future work should attempt to disentangle the relationship between income and age within the digital divide among Veterans. Furthermore, we found that older Veterans without Internet access were more likely to report being very uncomfortable using the Internet, suggesting that a lack of computer literacy could also contribute to the lack of access. This parallels studies of older civilians that found that older adult technology adoption is moderated by computer anxiety and confidence in computer skills [49,50]. In fact, even among the older Veterans who reported current Internet access, only 37% (54/147) reported being “very comfortable” using the Internet. Although some older Veterans may be able to access the Internet at home, they may still lack the confidence or skills to fully engage with HIT tools. Therefore, the potential for supported use through social ties is great even among those older Veterans who have opted for home Internet.

### The Potential For Social Access

We were particularly interested in the ability of older Veterans without Internet access to gain access through their social ties as this group exemplifies the “digital divide.” Encouragingly, our study revealed that even among this group, the majority still reported two or more social ties with home Internet access. These respondents also reported at least one adult child or extended family member who would use the Internet for the older Veteran. Previous research on older British civilians has similarly found that older adults might gain Internet access by using the computer of a family member [7,51]. Our work, the first to quantify potential social use of technology among US Veterans, corroborates these findings and contributes to the body of literature by examining Internet use of a vulnerable US population within the context of health management. Similarly, “surrogate” health information seeking, in which a friend or family member conducts an online search for the benefit of another, has been documented [52-54] and is likely quite commonplace. Studies of this activity have predominantly focused on identifying the characteristics and behaviors of “surrogate seekers,” who tend to be middle-aged, a spouse or parent, and serve as a caregiver [52-55]. Surrogate seekers also are more likely to engage in a variety of online content-generating activities, such as participating in online support groups or emailing health care providers [54]. Our study contributes to this literature by assessing the reported experiences and behaviors of older Veterans who may benefit from surrogate searches. Our older Veteran respondents appear to have multiple social ties who could perform a surrogate search for the benefit of health management, allowing the older adult to benefit from the Internet indirectly.

Those Veterans without Internet access also reported at least one adult child or extended family member that the older Veteran would feel comfortable asking for help to use the Internet. This corresponds with the concept of the “warm expert” whereby someone in a close relationship with the technology novice can serve as a mediator between the needs and skills of the novice and the technological system [56]. In other words, the family can provide instruction, assistance, and other instrumental support to the older adult for the purpose of direct technology access for health management. Recent polls suggest that 70% of older adults who currently use technology, and 87% of nonusers, say that they would need to ask someone for assistance to learn a new technology [6]. Thus, our finding that older Veterans report even a few social ties who could serve in this capacity suggests that older Veterans can identify the warm experts in their lives and may be willing to call on these social relationships for technology assistance. Interventions that educate families about the value of HIT for health management and promote skills for family technology collaboration may help to reduce the gap in older Veteran HIT use.

### Implications for Technological Design and Health Care Policy

Our study finds that older Veterans are able and willing to call on social ties for both direct and indirect access to the Internet. This indicates that social relationships may represent a possible solution to ensuring that older Veterans benefit from HIT innovations. However, one practical challenge to collaborative use of health technology concerns information privacy. For example, the majority of Americans desire to be in control of their personal information, both online and offline [57]. Therefore, older Veterans may not want to share their health information with their family or friends via collaborative use of HIT tools. Nevertheless, most of our participants endorsed comfort with having a social tie assist with Internet use in the context of health management, where health information is likely be transmitted. If an older Veteran feels such comfort asking their social tie for assistance, it is likely that the older Veteran would feel similar comfort with this close tie having access to their personal health information.

As another challenge, current technological design typically does not allow for social means of access, limiting the potential for social ties to engage and assist older users. For example, a scan of five prominent health care systems revealed that only two currently allow patients to designate a family member as a caregiver who can access all their personal health information. Thus, for most older patients, social ties are unable to assist with access to personal health information or providers (through electronic messaging) in a secure, confidential way. We recommend that developers design HIT tools that are conducive to multiple log-ins across multiple platforms in order to facilitate and encourage surrogate use and family collaboration. Similarly, we suggest that health care systems allow patients to delegate a surrogate who gains equal access to the HIT tools provided to patients. This could have a two-fold effect of encouraging family involvement in the care of older patients as well as enhancing information sharing between informal caregivers and family members.

### Limitations

Our study does have a few limitations. As noted, approximately 19% of our sampling cohort returned a completed survey, which is a lower response rate than we had targeted. As a result, we may be experiencing a nonresponse bias whereby those who returned the survey do not share the same characteristics as the entire sample. This may suggest that our findings do not represent most older Veterans. However, we found that our respondents are similar to the overall Veteran population across major demographic characteristics (ie, age, race, marital status, income, and education), and similar to those older adults most vulnerable to the digital divide: those with less education and income.

Additionally, our sample received the survey through the mail and could choose to participate by returning the completed survey. We may have experienced a participation bias, whereby those older Veterans who opted to return the survey were more comfortable with or interested in technology and more likely to have access. Yet, we found that almost half of our sample reported having no Internet access and that our sample reported engaging in technology at similar rates to the older civilian and overall Veteran populations. This could indicate that our findings accurately represent the wide spectrum of older Veteran use of technology and ability for access through social ties. In addition, we may have experienced an item nonresponse bias, whereby some older Veterans failed to respond to survey questions by mistake or purposefully. However, our study is not designed to represent a definitive scan of the population, but an initial inquiry in order to determine the feasibility of future social network interventions.

Finally, our study is not a traditional social network survey in that we investigate older Veteran reports of their social ties, but do not assess the social ties themselves. Although older Veterans may report the ability for direct and indirect Internet access through social ties, the social ties may account differently. Future work should examine the ability and willingness of family and friends to assist older adults with Internet access and use of HIT tools for a number of health management activities (eg, prescription requests vs bill payment vs access to clinical notes) in order to fully understand the experiences of all stakeholders.

### Conclusions

The digital divide puts some older adults at a disadvantage, limiting their ability to benefit from technology innovations that support health management. This study found that older Veterans are surrounded by social ties that do have access and can likely assist the older Veteran to use these tools. These findings can be used to design family interventions, develop HIT tools, and inform health care policy. In short, the potential for older Veteran access to HIT through social ties is great, and may serve as a partial solution to the digital divide.

## Abbreviations

HIT: health information technology
VHA: Veteran’s Health Administration
